# Oxidative stress links periodontal inflammation and renal function

**DOI:** 10.1111/jcpe.13414

**Published:** 2021-01-28

**Authors:** Praveen Sharma, Anthony Fenton, Irundika H. K. Dias, Brenda Heaton, Caroline L. R. Brown, Amneet Sidhu, Mutahir Rahman, Helen R. Griffiths, Paul Cockwell, Charles J. Ferro, Iain L. Chapple, Thomas Dietrich

**Affiliations:** ^1^ Periodontal Research Group School of Dentistry University of Birmingham and Birmingham Community Healthcare Trust Birmingham UK; ^2^ Department of Nephrology University Hospital Birmingham Birmingham UK; ^3^ Aston Medical School Aston University Birmingham UK; ^4^ Department of Health Policy and Health Services Research Boston University Henry M. Goldman School of Dental Medicine Boston MA USA; ^5^ Swansea University Singleton Park Swansea UK

**Keywords:** chronic kidney disease, inflammation, oxidative stress, periodontitis, structural equation modelling

## Abstract

**Aims:**

Patients with chronic kidney disease (CKD) are also susceptible to periodontitis. The causal link between periodontitis and CKD may be mediated via systemic inflammation/oxidative stress. Using structural equation modelling (SEM), this cross‐sectional study aimed to explore the causal relationship between periodontal inflammation (PI) and renal function.

**Materials and methods:**

Baseline data on 770 patients with stage 3–5 (pre‐dialysis) CKD from an ongoing cohort study were used. Detailed, bioclinical data on PI and renal function, as well as potential confounders and mediators of the relationship between the two, were collected. SEMs of increasing complexity were created to test the causal assumption that PI affects renal function and vice versa.

**Results:**

Structural equation modelling confirmed the assumption that PI and renal function are causally linked, mediated by systemic oxidative stress. The magnitude of this effect was such that a 10% increase in PI resulted in a 3.0% decrease in renal function and a 10% decrease in renal function resulted in a 25% increase in PI.

**Conclusions:**

Periodontal inflammation represents an occult source of oxidative stress in patients with CKD. Further clinical studies are needed to confirm whether periodontal therapy, as a non‐pharmacological approach to reducing systemic inflammatory/oxidative stress burden, can improve outcomes in CKD.

**Clinical Relevance**

*Scientific rationale for study*: Periodontitis is associated with chronic kidney disease (CKD). This study aimed to explore the causal mechanisms due to which these associations might occur.

*Principal findings*: Using structural equation modelling, this study confirmed the causal hypothesis that periodontitis and kidney function have a bidirectional relationship. This relationship is mediated via systemic oxidative stress.

*Practical implications*: It is yet to be determined whether the resolution of periodontal inflammation, via periodontal therapy, can influence the progression of CKD, and studies are underway to explore this. It remains an exciting, non‐pharmacological aid to the management of CKD.

## 1 INTRODUCTION

Chronic kidney disease (CKD) affects 8%–16% of the global population (Jha et al., 2013) and is strongly associated with premature mortality secondary to cardiovascular disease and progression to end‐stage kidney disease. Prognostic factors associated with worse outcomes in CKD include severity of kidney disease (Go et al., 2004), systemic inflammation and oxidative stress (Cachofeiro et al., 2008; Small et al., 2012). Periodontitis is a common chronic inflammatory disease affecting the connective tissues surrounding teeth. Severe periodontitis affects over 7% of the world's population (Kassebaum et al., 2017). Periodontal inflammation is associated with increased systemic inflammatory and oxidative stress (Allen et al., 2011; Tonetti & VanDyke, 2013).

The association between periodontitis and CKD has been highlighted (Kshirsagar et al., 2005; Franek et al., 2006; Craig, 2008). CKD is associated with a twofold increase in the prevalence of periodontitis (Deschamps‐Lenhardt et al., 2018). Periodontitis has been associated with increased all‐cause mortality in patients with CKD (Zhang et al., 2017). Periodontitis is also associated with worse outcomes in patients with cardiovascular disease (Dietrich et al., 2013) and diabetes (Sanz et al., 2018). Periodontitis treatment reduces plasma concentrations of pro‐inflammatory mediators (D'Aiuto et al., 2004, 2013; Freitas et al., 2012; Demmer et al., 2013; Teeuw et al., 2014) and, in patients with diabetes, reduces glycated haemoglobin (HbA1C) by 0.27%–0.48% (Sanz et al., 2018).

As inflammation and oxidative stress play a role in the pathobiology of periodontitis and CKD, these diseases may amplify adverse outcomes when occurring concomitantly (Deschamps‐Lenhardt et al., 2018). For example, periodontal inflammation could influence renal function through the dissemination of intact bacteria (Kshirsagar et al., 2007), bacterial products or pro‐inflammatory cytokines from inflamed, ulcerated periodontal tissues (Taylor et al., 2013; Tonetti & VanDyke, 2013; Cekici et al., 2014). CKD may affect periodontal inflammation via increases in systemic inflammatory and/or oxidative stress burden (Oberg et al., 2004; Cachofeiro et al., 2008; Small et al., 2012). It is also possible that associations between periodontal inflammation and renal function are artefactual, not causal.

Structural equation modelling (SEM) techniques elucidate complex biological relationships by integrating causal hypotheses in multiple, simultaneous regression models. This allows a variable to simultaneously be both an ‘exposure’ in one path and an ‘outcome’ in another. SEM can thus quantify the direct and indirect effect of one variable on another.

In this study, we utilized SEM to assess a well‐characterized cohort of patients with CKD from an established cohort study. We aimed to assess the effect of periodontal inflammation on renal function and vice versa, and how these effects may be mediated.

## 2 MATERIALS AND METHODS

Secondary analysis was carried out using data derived from baseline assessments in a prospective cohort study, the Renal Impairment In Secondary Care (RIISC) study (Stringer et al., 2013). Briefly, between October 2010 and December 2015 the RIISC study recruited a cohort of patients with CKD who were at high risk of adverse outcomes.

Participants had been under follow‐up in a secondary care renal clinic for at least 1 year prior to recruitment and fulfilled 2008 National Institute of Clinical Excellence CKD guidelines for referral to a specialist service for management of CKD. Inclusion criteria comprised either stage 3 CKD with an estimated glomerular filtration rate (eGFR) decline ≥5 mls/min/year or ≥10 mls/min/5 years; or stage 4 or 5 CKD (pre‐dialysis); or a urine albumin creatinine ration (ACR)>70 mg/mmol on three occasions. Patients undergoing renal replacement therapy (dialysis or kidney transplant) or receiving treatment with immunosuppressive medication were excluded. The study was approved by the South Birmingham Local Research Ethics Committee (ref:17010/H1207/6) and University Hospitals Birmingham Research and Development Department (ref:RRK3917). It was conducted following the principles of the Declaration of Helsinki with participants giving written informed consent. The study is registered on clinicaltrials.gov (ref:NCT01722383). STROBE (Strengthening the Reporting of Observational Studies in Epidemiology) guidance was used in the generation of this manuscript.

### 2.1 Renal assessments

The CKD‐EPI (Chronic Kidney Disease Epidemiology Collaboration) equation incorporating creatinine was used to calculate eGFR (Levey et al., 2009). Serum creatinine was measured using a Roche Modular Analyser using a blank rated compensated Jaffe reaction. Albuminuria was assessed using urine ACR, which was measured using the ADVIA 1800 Chemistry System (Bayer HealthCare).

### 2.2 Periodontal assessments

Participants underwent a full‐mouth, detailed periodontal assessment, recording periodontal probing depths (PPD), gingival recession and clinical attachment loss at interproximal sites of all teeth present. Bleeding on probing (BOP) was recorded at each site (present/absent). The PPD and BOP data were used to calculate the periodontal inflamed surface area (PISA) (Nesse et al., 2008), which is an estimate of the area (in mm^2^) of the inflamed periodontal tissue. To aid description of this cohort, cases of periodontitis were defined using the Page & Eke, 2007 classification (Page & Eke, 2007) and mean PPD, CAL and BOP were calculated.

### 2.3 Assessment of inflammation and oxidative stress

C‐reactive protein (CRP) was measured from serum samples using the Full Range CRP Kit (range from 0.2 to 400 mg/L) (The Binding Site Group Ltd). The polyclonal serum‐free light chain (sFLC) concentration, a measure of systemic humoral response, was the summation of κ and λ sFLC concentrations obtained using the Dade‐Behring Nephelometer II (BNII) Analyzer System (Siemens AG) and particle‐enhanced high‐specificity homogenous immunoassays (Freelite; The Binding Site Group Ltd).

Systemic oxidative stress was evaluated by measurement of protein carbonyls (protein oxidation) and F2‐α‐isoprostanes (lipid peroxidation). Protein carbonyls were assessed by ELISA following the method of Carty et al., 2000. 8‐isoprostane F2α levels were measured by EIA method according to the manufacturer's instructions (Cayman Chemicals).

### 2.4 Demographic and other laboratory variables

Patient age in years, sex (male/female), ethnicity (White or non‐White), smoking status (current/former/never), diabetes (yes/no), employment status (yes/no) and educational attainment were ascertained. Measurements of body height and weight were carried out to determine body mass index (BMI), and venous blood samples were taken to assess HbA1C and serum concentrations of calcium, phosphate and bicarbonate. Blood pressure (BP) was measured using the BpTRU automated device (BpTRU Medical Devices), which has been reported to be comparable to the mean daytime BP from 24‐hour ambulatory BP monitoring (Brothwell et al., 2013). Hypertension, defined as use of antihypertensive medication or average systolic BP ≥ 140 mmHg or average diastolic BP ≥ 90 mmHg, was used in the analyses. Formation of advanced glycation end products (AGEs) is accelerated by increased systemic glucose levels (Yan et al., 2008). The deposition of AGEs in skin was measured using skin autofluorescence (AGE reader TM; Diagnoptics Technologies).

### 2.5 Statistical analysis

**FIGURE 1:**
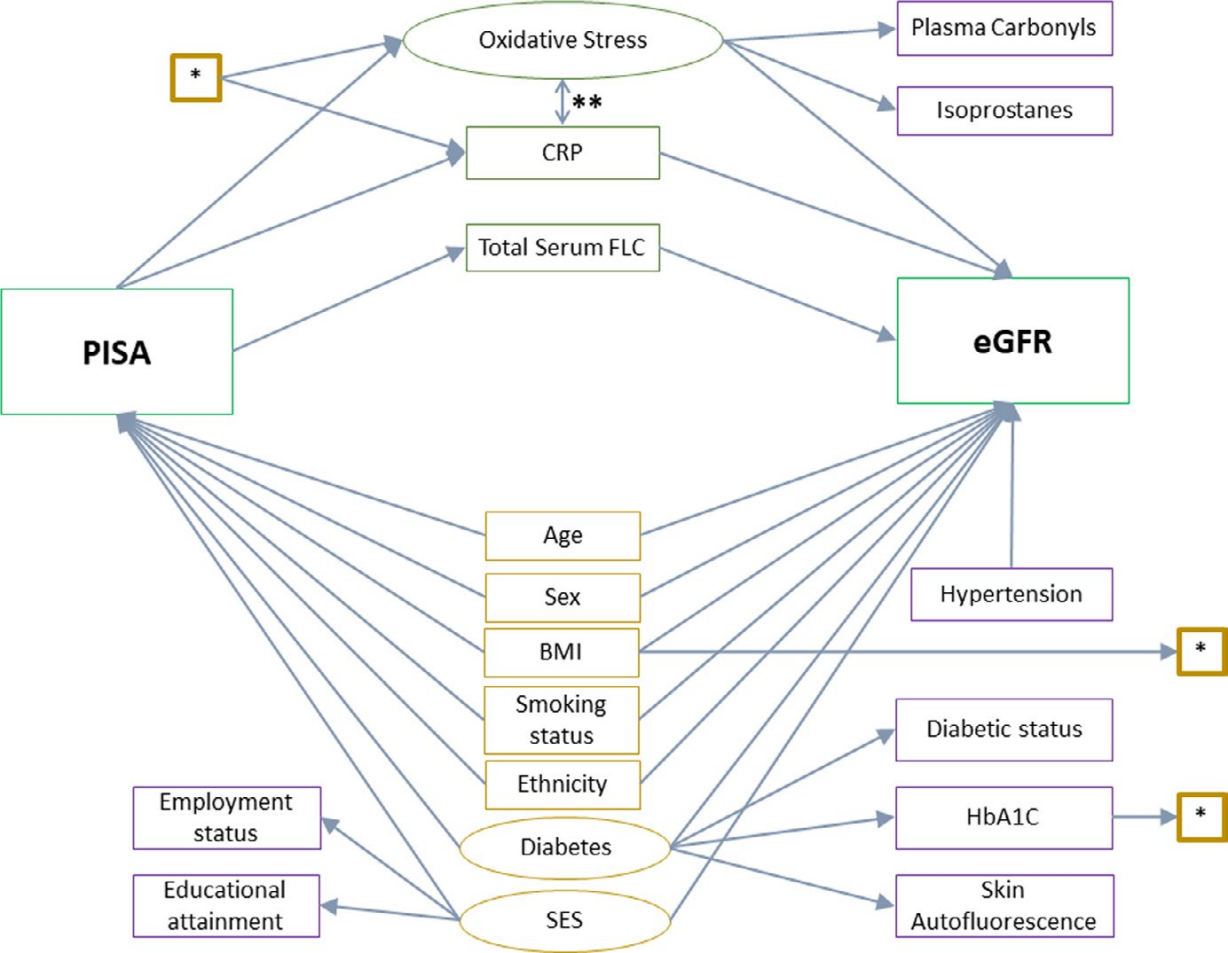
Causal diagram for the structural equation model with renal function, eGFR, as the outcome. Rectangles: observed variables; ovals: latent variable; green: exposure and outcomes of interest; purple: confounders; orange: mediators. *—paths included in Model 2, in addition to the base model. **—paths included in Model 3, in addition to Model 2. BMI, body mass index; CRP, c‐reactive protein; eGFR, estimated glomerular filtration rate; FLC, free light chain; HbA1C, glycated haemoglobin A1C; PISA, periodontal inflamed surface area; SES, socio‐economic status

**FIGURE 2:**
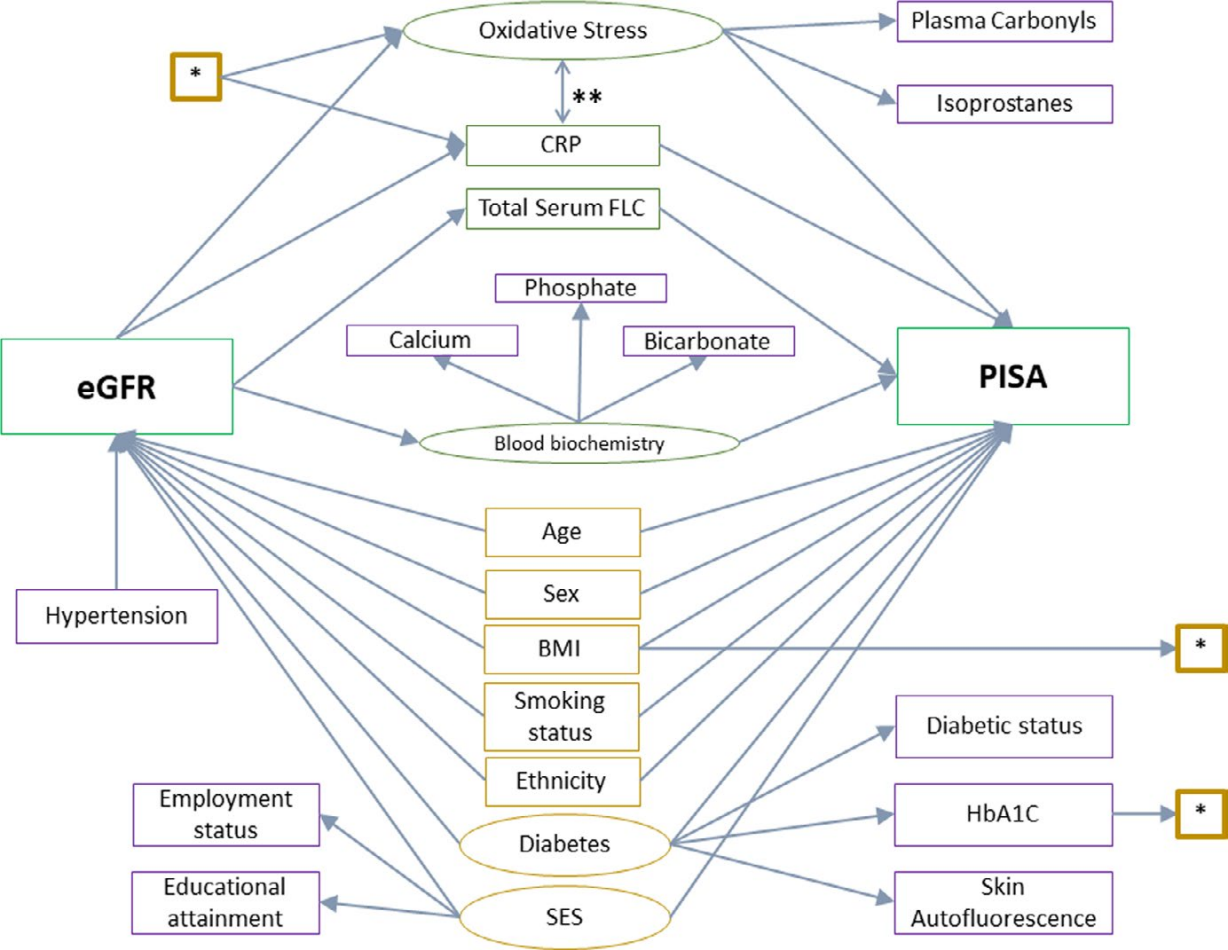
Causal diagram for the structural equation modelling with periodontal inflammation, PISA, as the outcome. Rectangles: observed variables; ovals: latent variable; green: exposure and outcomes of interest; purple: confounders; orange: mediators. *—paths included in Model 2, in addition to the base model. **—paths included in Model 3, in addition to Model 2. BMI, body mass index; CRP, c‐reactive protein; eGFR, estimated glomerular filtration rate; FLC, free light chain; HbA1C, glycated haemoglobin A1C; PISA, periodontal inflamed surface area; SES, socio‐economic status

To test proposed causal assumptions, we specified SEMs based on causal diagrams (Figures 1 and 2). Causal diagrams take a structural approach to assessing the potential for confounding and mediation (Greenland et al., 1999; Heaton & Dietrich, 2012). SEMs were used to estimate the direct effect of all predictive variables on outcomes of interest. In addition to the base model (model 1, Figures 1 and 2), models of increasing complexity were considered. Model 2 (* in Figures 1 and 2) included HbA1C and BMI as additional causal determinants of systemic CRP and oxidative stress levels. Model 3 (** in Figures 1 and 2) captured the interplay between CRP and oxidative stress by adding a feedback loop between these.

Latent variables for oxidative stress, diabetes and SES, were obtained following discussions with the authors as to the causal relationship between the latent variables and the observed variables that load onto them. The standardized direct effects were estimated using maximum likelihood with missing values to account for missing data. All analyses were carried out using Stata/IC version 15.1 (StataCorp LLC). Robust estimations of the standard errors were generated using STATA's ‘vce (robust)’ command with a 2‐sided significance level of 0.05. The unstandardized indirect effects were calculated using the products of the path coefficients along the indirect path between exposure and outcome. Direct and indirect effects were presented, along with the 95% confidence interval. The in/direct effects are interpreted as the effect on the outcome for a 1‐unit change in the exposure. Here, the ‘unit’ is scaled to a percentage; hence, the coefficients are interpreted as the percentage change in outcome for a 10% change in exposure. Details of model development are included in a Supplementary File.

## 3 RESULTS

### 3.1 Study population

Between October 2010 and December 2015, 770 participants were recruited into the RIISC study at the centre where periodontal assessment was being done. 93.6% (*n* = 721) of participants underwent a detailed periodontal examination. Of these, 15% (108) were edentulous and were excluded from further analysis yielding a final sample size of 613. Of these, 5% (*n* = 30) were periodontally healthy, 47% (*n* = 287) had moderate periodontitis, and 48% (*n* = 296) had severe periodontitis (Page & Eke, 2007). The mean eGFR for the sample was 37 ml/min/1.73 m^2^, mean age was 61 years; 62% were male, 67.6% were White, 50% were never‐smokers, and 36% had diabetes. The mean PISA for this group was 483 mm^2^ (Table 1).

**TABLE 1:** Baseline demographics expressed as mean (SD), unless otherwise stated

	Whole cohort (*N* = 721)	% missing data	Mild/no periodontitis (*n* = 30)	Moderate periodontitis (*n* = 287)	Severe periodontitis (*n* = 296)
Age (years)	61 (16)	0	50 (18)	59 (17)	64 (14)
Male (%)	62	0	47	60	65
Ethnicity (%)					
White	67.6	0	73.3	70.4	64.2
South Asian	22.4		13.3	19.2	26.7
Black	9.3		13.3	9.4	8.8
Other	0.7		0	1.1	0.3
Smoker (%)					
Never	50.2	1.8	66.7	57.8	43.0
Former	34.5		20.0	33.0	38.8
Current	13.5		13.3	9.2	9.2
Diabetic (%)	36	0	30	30	42
HbA1C (mmols/mol)	49 (17.4)	4.7	46 (19.7)	47 (15.8)	51 (18.6)
BMI (kg/m^2^)	30 (7)	3.3	29 (6)	30 (7)	30 (7)
eGFR (ml/min/1.73 m^2^)	37 (20)	1.95	47 (25)	38 (21)	35 (19)
uACR (mg/mmol)	100 (144)	10	151 (190)	87 (131)	108 (150)
Haemoglobin (g/dl)	12.4 (1.7)	1.8	12.7 (21)	12.3 (1.9)	12.5 (1.6)
Phosphate (mmol/L)	1.1 (0.2)	2.0	1.1 (0.2)	1.1 (0.2)	1.2 (0.2)
Calcium (mmol/L)	2.2 (0.1)	1.6	2.3 (0.1)	2.3 (0.1)	2.2 (0.1)
Bicarbonate (mEq/L)	24 (3.3)	2.4	24 (3.2)	24 (3.3)	24 (3.3)
Total cholesterol (mmol/L)	4.8 (1.4)	1.8	5.3 (1.8)	4.9 (1.3)	4.7 (1.4)
Hypertension (%)	94	0.5	93	94	94
PISA (mm^2^)	483 (532)	0.33	105 (114)	349 (360)	652 (632)
Mean PPD (mm)	2.7 (0.8)	0	1.8 (0.5)	2.5 (0.5)	3.4 (0.8)
Mean CAL (mm)	3.6 (1.5)	0	2.4 (2.2)	2.8 (0.7)	4.4 (1.4)
BOP (%)	33.8 (24.4)	0	12.8 (11.7)	26.8 (20.0)	42.6 (25.7)
CRP (mg/L)	7.4 (12.8)	0.8	6.0 (10.3)	7.5 (12.7)	7.5 (13.2)
Total serum FLC concentration	108 (187)	0.8	74 (38)	109 (162)	112 (218)
Isoprostane (pg/ml)	26 (20)	26.8	22 (15)	26 (21)	26 (19)
Protein carbonyls (nmol/mg of protein)	1.2 (0.7)	25.9	1.1 (0.7)	1.1 (0.6)	1.2 (0.7)
Currently employed (%)	33	0.5	50	37	28
Highest educational qualification (%)					
None	40	1.6	23	34	47
GCSE	24		27	25	23
NVQ	8		3	8	9
GCE A‐Level	9		10	10	7
UG	13		30	14	10
PG	7		7	9	5

Abbreviations: GCE A‐Level, General Certificate of Education Advanced Level (aged approximately 18); GCSE, General Certificate of Secondary Education (aged approximately 16); NVQ, National Vocal Qualification (aged approximately 16–18); PG, postgraduate; UG, undergraduate.

### 3.2 Structural equation modelling

#### 3.2.1 Model investigating the effect of PISA on eGFR

The base model (Figure 1, model 1) with eGFR as the outcome and PISA as the exposure of interest showed no clinically or statistically significant direct effect of PISA on total serum FLC or CRP concentrations. There was a clinically and statistically significant direct effect of PISA on the latent variable oxidative stress with a 0.21 SD (95% CI: 0.06 to 0.36) change in the latent variable oxidative stress seen for every one SD increase in PISA. There was a clinically and statistically significant direct effect of total serum FLC concentration and the latent variable oxidative stress on eGFR with a 0.49 SD (95% CI: 0.42 to 0.56) and a 0.33 SD (95% CI: 0.20 to 0.45) change in eGFR seen with a one SD increase in serum FLC and the latent variable oxidative stress, respectively. There was no clinically or statistically significant direct effect of CRP on eGFR (Figure 1; Table 2).

**TABLE 2:** Direct (standardized) and indirect (unstandardized) effects from PISA to eGFR

Paths	Model 1		Model 2 (model 1 + HbA1C and BMI as confounders of exposure‐>mediator and mediator‐>outcome paths)		Model 3 (model 2 + feedback loop between CRP and OS)	
	Path coefficient (95% CI)	*p*‐Value	Path coefficient (95% CI)	*p*‐Value	Path coefficient (95%CI)	*p*‐Value
Direct						
PISA‐>sFLC	0.002 (−0.077 to 0.081)	0.959	0.002 (−0.077 to 0.081)	0.962	0.002 (−0.077 to 0.081)	0.960
PISA‐>CRP	−0.073 (−0.150 to 0.004)	0.063	−0.074 (−0.149 to 0.001)	0.052	−0.049 (−0.125 to 0.028)	0.212
PISA‐>OS	−0.212 (−0.361 to −0.062)	0.006	−0.210 (−0.363 to −0.057)	0.007	−0.212 (−0.369 to −0.057)	0.008
sFLC‐>eGFR	−0.490 (−0.563 to −0.416)	<0.001	−0.489 (−0.563 to −0.415)	<0.001	−0.489 (−0.564 to −0.415)	<0.001
CRP‐>eGFR	−0.028 (−0.098 to 0.042)	0.428	−0.029 (−0.099 to 0.041)	0.419	−0.055 (−0.138 to 0.026)	0.182
OS‐>eGFR	0.327 (0.204 to 0.451)	<0.001	0.330 (0.208 to 0.452)	<0.001	0.334 (0.199 to 0.468)	<0.001
HbA1C‐>CRP			0.165 (0.085 to 0.244)	<0.001	0.166 (0.087 to 0.246)	<0.001
BMI‐>CRP			0.226 (0.150 to 0.303)	<0.001	0.221 (0.142 to 0.300)	<0.001
HbA1C‐>OS			−0.006 (−0.205 to 0.193)	0.954	−0.010 (−0.227 to 0.208)	0.929
BMI‐>OS			0.045 (−0.149 to 0.238)	0.650	0.048 (−0.152 to 0.249)	0.637
OS‐>CRP					0.119 (0.022 to 0.217)	0.017
CRP‐>OS					−0.019 (−0.224 to 0.185)	0.853
Indirect						
PISA‐‐>sFLC‐‐> eGFR	0.000 (−0.015 to 0.015)	0.959	0.000 (−0.015 to 0.015)	0.962	0.000 (−0.015 to 0.015)	0.960
PISA‐‐>OS‐‐> eGFR	−0.027 (−0.047 to −0.006)	0.010	−0.027 (−0.048 to −0.006)	0.012	−0.027 (−0.049 to −0.006)	0.014
PISA‐‐>CRP‐‐> eGFR	0.001 (−0.001 to 0.003)	0.465	0.001 (−0.001 to 0.003)	0.453	0.001 (−0.001 to 0.003)	0.348
PISA‐‐>OS‐‐> CRP‐‐>eGFR					0.001 (−0.001 to 0.001)	0.270
PISA‐‐>OS‐‐> CRP‐‐>OS‐‐>eGFR					0.000 (0.000 to 0.000)	0.863
PISA‐‐>CRP‐‐> OS‐‐>eGFR					0.000 (−0.001 to 0.001)	0.853
PISA‐‐>CRP‐‐> OS‐‐>CRP‐‐>eGFR					0.000 (0.000 to 0.000)	0.853

Note

NB: the latent variable OS (oxidative stress) is generated with lower values representing higher levels of oxidative stress.

Abbreviations: BMI, body mass index; CRP, c‐reactive protein; eGFR, estimated glomerular filtration rate; HbA1C, glycated haemoglobin A1C; OS, oxidative stress; PISA, periodontal inflamed surface area; sFLC, serum‐free light chain.

The next model (model 2) showed a clinically and statistically significant effect of BMI and HbA1C on CRP but not oxidative stress. The point estimates and confidence intervals from the previous model were stable to these changes (Table 2).

The final model (model 3) showed no appreciable clinically or statistically significant effect of CRP on the latent variable oxidative stress. There was a clinically significant effect of the latent variable oxidative stress on CRP with a 1 SD increase in the latent variable oxidative stress leading to a 0.12 SD (95% CI: −0.02 to 0.21) change in CRP, but this was not statistically significant. The point estimates and confidence intervals from the previous model were stable to these changes (Table 2).

In investigating the indirect, path‐specific effect of PISA on eGFR, there was no clinically or statistically significant effect of PISA on eGFR mediated via changes in serum FLC or CRP concentrations. There was a clinically and statistically significant indirect effect of PISA on eGFR, mediated via oxidative stress, such that a 10% increase in PISA led to a 3.0% decrease in eGFR (95% CI: 0.6–5.4) (Table 2).

#### 3.2.2 Model investigating the effect of eGFR on PISA

The base model (Figure 2, model 1) with PISA as the outcome and eGFR as the exposure of interest showed that eGFR has a clinically and statistically significant direct effect on serum FLC, CRP concentration and the latent variables oxidative stress and blood biochemistry with a 0.54 SD (95% CI: 0.48 to 0.60), a 0.17 SD (95% CI: 0.09 to 0.25), a 0.40 SD (95% CI: 0.26 to 0.54) and a 0.64 SD (95% CI: 0.51 to 0.77) change seen respectively for every one SD decrease in eGFR. Serum FLC and CRP concentration and the latent variable blood biochemistry were not shown to have a clinically or statistically significant effect on PISA. The latent variable oxidative stress had a clinically significant direct effect on PISA, but this was not statistically significant (Figure 2; Table 3).

**TABLE 3:** Direct (standardized) and indirect (unstandardized) effects from eGFR to PISA

Paths	Model 1		Model 2 (model 1 + HbA1C and BMI as confounders of exposure‐>mediator and mediator‐>outcome paths)		Model 3 (model 2 + feedback loop between CRP and OS)	
	Path coefficient (95% CI)	*p*‐Value	Path coefficient (95% CI)	*p*‐Value	Path coefficient (95% CI)	*p*‐Value
Direct						
eGFR‐>sFLC	−0.540 (−0.598 to −0.483)	<0.001	−0.540 (−0.598 to −0.483)	<0.001	−0.540 (−0.597 to −0.483)	<0.001
eGFR‐>CRP	−0.171 (−0.248 to −0.093)	<0.001	−0.169 (−0.241 to −0.097)	<0.001	−0.118 (−0.193 to −0.043)	0.002
eGFR‐>OS	−0.401 (−0.539 to −0.263)	<0.001	−0.392 (−0.546 to −0.238)	<0.001	−0.468 (−0.599 to −0.336)	<0.001
eGFR‐>BB	0.639 (0.506 to 0.773)	<0.001	0.640 (0.506 to 0.774)	<0.001	0.639 (0.506 to 0.772)	<0.001
CRP‐>PISA	−0.020 (−0.099 to 0.058)	0.611	−0.020 (−0.099 to 0.059)	0.621	0.010 (−0.079 to 0.099)	0.829
sFLC‐>PISA	−0.012 (−0.104 to 0.080)	0.800	−0.012 (−0.104 to 0.080)	0.801	0.000 (−0.089 to 0.088)	0.995
OS‐>PISA	0.155 (−0.032 to 0.343)	0.105	0.148 (−0.049 to 0.346)	0.141	0.195 (0.034 to 0.356)	0.017
BB‐>PISA	−0.063 (−0.215 to 0.088)	0.412	−0.068 (−0.224 to 0.087)	0.389	0.000 (0.000 to 0.000)	<0.001
HbA1C‐>CRP			0.154 (0.076 to 0.233)	<0.001	0.151 (0.066 to 0.236)	<0.001
BMI‐>CRP			0.233 (0.158 to 0.307)	<0.001	0.237 (0.157 to 0.317)	<0.001
HbA1C‐>OS			0.025 (−0.149 to 0.199)	0.780	0.075 (−0.110 to 0.259)	0.426
BMI‐>OS			−0.045 (−0.203 to 0.114)	0.580	0.039 (−0.129 to 0.207)	0.651
OS‐>CRP					0.122 (0.074 to 0.170)	<0.001
CRP‐>OS					−0.315 (−0.456 to −0.174)	<0.001
Indirect						
eGFR‐‐>sFLC‐‐> PISA	0.016 (−0.108 to 0.140)	0.801	0.016 (−0.108 to 0.140)	0.802	0.000 (−0.118 to 0.119)	0.995
eGFR‐‐>OS‐‐> PISA	−0.155 (−0.367 to 0.057)	0.151	−0.145 (−0.371 to 0.081)	0.209	−0.228 (−0.451 to −0.004)	0.046
eGFR‐‐>CRP‐‐> PISA	0.009 (−0.025 to 0.043)	0.616	0.008 (−0.025 to 0.042)	0.626	−0.003 (−0.029 to 0.023)	0.829
eGFR‐‐>BB‐‐> PISA	−0.101 (−0.345 to 0.143)	0. 417	−0.109 (−0.360 to 0.142)	0.395	0.000 (0.000 to 0.000)	<0.001
eGFR‐‐>OS‐‐> CRP‐‐>PISA					−0.001 (−0.014 to 0.011)	0.831
eGFR‐‐>OS‐‐> CRP‐‐>OS‐‐>PISA					0.000 (−0.000 to 0.001)	0.834
eGFR‐‐>CRP‐‐> OS‐‐>PISA					0.018 (−0.003 to 0.039)	0.088
eGFR‐‐>CRP‐‐> OS‐‐>CRP‐‐>PISA					0.000 (−0.001 to 0.001)	0.832

Abbreviations: BB, blood biochemistry; BMI, body mass index; CRP, c‐reactive protein; eGFR, estimated glomerular filtration rate; HbA1C, glycated haemoglobin A1C; OS, oxidative stress; PISA, periodontal inflamed surface area; sFLC, serum‐free light chain.

The next model (model 2) showed a clinically and statistically significant effect of BMI and HbA1C on CRP but not oxidative stress. The point estimates and confidence intervals from the previous model were stable to these changes (Table 3).

The final model (model 3) showed a clinically and statistically significant effect of CRP on the latent variable oxidative stress and vice versa. The point estimates and confidence intervals from the previous model were stable to these changes (Table 3).

In investigating the path‐specific effect of eGFR on PISA, there was no clinically or statistically significant effect of eGFR on PISA via any pathway, apart from via the latent variable oxidative stress where a 10% decrease in eGFR led to a 25.0% increase in PISA (95% CI: 0.4 to 49.6).

## 4 DISCUSSION

We found a bidirectional, causal relationship between periodontal inflammation and renal function: a 10% increase in PISA led to a 3.0% decrease in eGFR and a 10% decrease in eGFR led to a 25.0% increase in PISA. The 10% change in PISA in this cohort can be contextualized by comparing mean PISA in healthy/mild periodontitis patients (105 mm^2^), moderate periodontitis (349 mm^2^) and severe periodontitis (652 mm^2^) (Table 1). Based on the 4‐variable, 5‐year risk of kidney failure equation for non‐American populations (Tangri et al., 2016), a 3% decrease in eGFR would, in this cohort, translate to a change in 5‐year risk of kidney failure from 32% (SD 29%) to 34% (SD 30%). We also showed that oxidative stress provided the pathobiological basis for this bidirectional relationship, not the inflammatory load as measured by CRP and sFLCs, or altered blood biochemistry measured by calcium, phosphate and bicarbonate levels.

This study is the largest of its kind with detailed periodontal phenotyping of patients with CKD at an elevated risk of CKD progression. Collection of detailed demographic and bioclinical data allowed for adjustment of factors that might confound the relationship between periodontal inflammation, renal function and systemic inflammatory/oxidative stress markers. Oxidative stress was measured using assays validated in multicentre method validation studies (Breusing et al., 2010; Augustyniak et al., 2015). The clinical and laboratory assessments allowed for comprehensive testing of causal assumptions using SEM. SEM facilitated the examination of many associations simultaneously and assumptions regarding the bidirectional effect of periodontal inflammation on renal function. The direct effects were standardized to allow comparison of the magnitude of various direct effects. These were interpreted as a per cent change in the SD in the outcome for a 1 SD change in the exposure. To aid clinical interpretation, the indirect effects were not standardized and were interpreted as a per cent change in the outcome for a 10% change in the exposure.

We also used ‘latent variables’ in addition to ‘observed variables’ in SEM. Latent variables allow researchers to quantify unmeasured or unmeasurable confounders using data on observed variables associated with them. For example, ‘socio‐economic status’ (SES) is notoriously difficult to quantify. Using SEM, a latent variable for SES can be constructed based on observed variables such as employment status and highest educational attainment. SEM therefore has the potential to allow detailed assessment of the pathobiological relationship between periodontitis and CKD. Another strength of this study is the use of PISA to quantify periodontal inflammation. The use of case definitions of periodontitis, designed for epidemiological purposes, in investigating the association between periodontitis and systemic diseases, has previously been criticized (Ioannidou et al., 2010; Grubbs et al., 2016) as it may not represent current periodontal health/inflammatory status. As a measure of periodontal inflammatory load, PISA represents a close approximation of the underlying causal exposure. The choice of measure of periodontal disease is important when unravelling the causal pathways between periodontal and systemic diseases.

This study builds on previous work evaluating the association between renal function and periodontal health (Ariyamuthu et al., 2013; Chambrone et al., 2013; Deschamps‐Lenhardt et al., 2018; Zhao et al., 2018). Previous studies referred to a lack of evidence for a causal link between periodontitis and CKD (Nanayakkara & Zhou, 2019). A previous study using SEM on data from the National Health and Nutrition Examination Study (NHANES) III demonstrated a bidirectional relationship between periodontitis and CKD (Fisher et al., 2011). We also reported a bidirectional relationship using more detailed periodontal and bioclinical phenotyping of patients and more robust testing of the possible causal mechanisms. Another study used causal mediation analysis on data from the Electric Generation Authority of Thailand study to show a significant direct and indirect (via diabetes) effect of periodontitis on the incidence of CKD (Lertpimonchai et al., 2019). This study lacked data on inflammatory and oxidative stress markers and hence was unable to elucidate the mechanism of effect. Therefore, our study advances the understanding of how periodontal inflammation and renal function influence each other by demonstrating the mediating role of systemic oxidative stress. Our findings align with animal studies showing renal tissue damage linked to oxidative stress following periodontitis (Franca et al., 2017).

Our study showed no major effect of periodontal inflammation on serum FLC concentration, contrary to a prior study (White et al., 2016). This may be due to differences in periodontitis assessment or patient population; the previous study was conducted in participants with normal renal function and therefore normal renal clearance of FLCs. This may indicate that the increase in serum FLC concentration seen with decreased renal clearance of FLCs far outweighs the contribution of periodontal inflammation to a rise in serum FLC concentration. Our study found no evidence of a significant effect of periodontal inflammation on systemic CRP levels contradicting previous reports and reviews (D'Aiuto et al., 2013; Demmer et al., 2013; Kumar et al., 2013). These discrepant findings may be due to the substantially higher levels of CRP in patients with CKD, consistent with other reports of high CRP levels in patients with CKD (Kalantar‐Zadeh, 2007). Hence, our study may be underpowered to detect the increases in CRP reported in other cohorts of patients with periodontitis (Demmer et al., 2013). Patients with CKD have a greater relative increase in CRP as compared to protein carbonyls or isoprostanes (Oberg et al., 2004). In patients with CKD, the less dramatic increase in markers of oxidative stress, compared with CRP, may make it possible for the effects of periodontal inflammation to be more readily detected in changes in oxidative stress levels than in changes in CRP levels.

Our study also has limitations. Firstly, we are limited by the assumptions made in the causal diagrams describing the relationships between periodontal inflammation and renal function (Figures 1 and 2). The presence and absence of variables and arrows, along with the direction of arrows, are assumptions based on current thinking. The comprehensive causal diagrams do not capture the full complexity of the biological interactions. There may be an effect of renal function on periodontal inflammation, via the effect of renal function on immune function. In the absence of data quantifying immune function, this analysis was not possible. Furthermore, the cross‐sectional nature of this study does not allow for investigations of temporality, which limits the understanding of the true causal nature of the observed relationships. Also, as with observational studies in general, unmeasured confounding, such as the effect of poor diet on both periodontal health and renal function, may explain the observed relationship.

Additionally, analyses of isoprostanes and plasma carbonyls were only carried out on the first 586 participants, resulting in missing data for the remainder of the cohort. This was mitigated by imputing the missing data using STATA's maximum likelihood with missing values option in the SEMs. This adjusts the likelihood function, based on Markov chain Monte Carlo estimations, to allow every case to contribute information on the observed variables. In addition, sensitivity analyses conducted, limiting the data to those with known values of isoprostanes and plasma carbonyls, yielded similar results (data not shown). Finally, the particularly high CRP levels in this cohort, which may reflect the progressive nature of their CKD, may limit the generalizability of these results.

Our study highlights the role of periodontal inflammation as an occult source of increased systemic oxidative stress in CKD. Collecting data on oxidative stress in future studies may expand on these results. It is not known whether periodontitis treatment will improve the oxidative stress burden in patients with CKD and, ultimately, impact morbidity and mortality associated with CKD. Pilot studies are underway, and these may provide the basis for randomized control trials to confirm whether the mechanistic link reported here is modifiable through periodontitis treatment and whether this improves clinical outcomes in patients with CKD.

## ETHICAL STATEMENT

The study was approved by the South Birmingham Local Research Ethics Committee (ref:17010/H1207/6) and University Hospitals Birmingham Research and Development Department (ref:RRK3917). It was conducted following the principles of the Declaration of Helsinki with participants giving written informed consent. The study is registered on clinicaltrials.gov (ref:NCT01722383). STROBE (Strengthening the Reporting of Observational Studies in Epidemiology) guidance was used in the generation of this manuscript.

## CONFLICT OF INTEREST

The authors declare no conflict of interest regarding this work.

## AUTHOR CONTRIBUTIONS

PS, AS and MR were responsible for the oral examinations of participants; AF, PC and CJF were responsible for the renal and medical assessments of participants; ILC, IHKD and HRG were responsible for the conceptualization of the oxidative stress measurement assays; IHKD and CLRB were responsible for running these assays; PS and BH undertook the statistical analysis; PC, ILC and TD designed the study; PS drafted and revised the manuscript. All authors gave their final approval and agreed to be accountable for all aspects of the work.

## Supporting information

Supplementary Material

## Data Availability

Pseudonymized individual participant data, used in preparation for this manuscript, will be available immediately following publication for a period of 24 months. This will be available to researchers providing a methodologically sound proposal and for the purposes of achieving the aims of that proposal only. Proposals should be directed to the corresponding author. To gain access, researchers will need to sign a data access agreement.
