# Supplementary material for: Oxidative stress links periodontal inflammation and renal function
Source: J Clin Periodontol. 2021 Jan 28;48(3):357–67. doi: 10.1111/jcpe.13414 (PMC7986430; doi:10.1111/jcpe.13414)
Supplement: Supplementary file 1 — Supplementary Material [file JCPE-48-357-s001.docx]

TITLE: Oxidative stress links periodontal inflammation and renal function

AUTHORS: Praveen Sharma, Anthony Fenton, Irundika HK Dias, Brenda Heaton, Caroline LR Brown, Amneet Sidhu, Mutahir Rahman, Helen R. Griffiths, Paul Cockwell, Charles J. Ferro, Iain L. Chapple, Thomas Dietrich

**Supplementary material**

**Supplemental Methods**

*Model Development*

-Model specification.

The models were based on discussion between the authors of the biology underpinning the presence and direction of relationships between variables, as opposed to a statistical threshold for choosing the model. Examination of endogenous variables revealed most to have a log-normal distribution. These were log-transformed to aid the assumption of multi-variate normality.

-Model fit*.*

The overall goodness of fit of the models was assessed and verified using the coefficient of determination (CD) with values >0.9 indicating good overall fit of the model. As Model 3 (** in Figures 1 & 2) was non-recursive, the stability of the models was tested using an Eigenvalue stability index of <1 as an indicator of a stable model.

**Supplemental tables**

Supplemental Table 1: Baseline demographics (expressed as mean (SD), unless otherwise stated) by Stage of periodontitis based on CAL alone (Tonetti, Greenwell et al. 2018).

|  | Whole cohort  (N=613) | % missing data | Stage II periodontitis  (n=64) | Stage III/IV periodontitis (n=549) |
| --- | --- | --- | --- | --- |
| **Age** | 61 (16) | 0 | 50 (17) | 62 (15) |
| **Male** | 62% | 0 | 45% | 64% |
| **White** **Ethnicity** | 67.6% | 0 | 67% | 68% |
| **Smoker**  Never  Former  Current | 50.2%  34.5%  13.5% | 1.8 | 71.4%  19.1%  9.5% | 48.8%  36.9%  14.3% |
| **Diabetic** | 36% | 0 | 22% | 34% |
| **HbA1C (mmols/mol)** | 49 (17.4) | 4.7 | 45 (19.1) | 49 (17.2) |
| **BMI (kg/m^2^)** | 30 (7) | 3.3 | 29 (7) | 30 (7) |
| **eGFR (ml/min/1.73m^2^)** | 37 (20) | 1.95 | 45 (25) | 36 (19) |
| **uACR (mg/mmol)** | 100 (144) | 10 | 78 (98) | 102 (145) |
| **Haemoglobin (g/dl)** | 12.4 (1.7) | 1.8 | 12.4 (1.8) | 12.4 (1.7) |
| **Phosphate (mmol/L)** | 1.1 (0.2) | 2.0 | 1.1 (0.2) | 1.1 (0.2) |
| **Calcium (mmol/L)** | 2.2 (0.1) | 1.6 | 2.3 (0.1) | 2.2 (0.1) |
| **Bicarbonate (mEq/L)** | 24 (3.3) | 2.4 | 24 (2.8) | 24 (3.3) |
| **Total cholesterol (mmol/L)** | 4.8 (1.4) | 1.8 | 5.2 (1.6) | 4.8 (1.3) |
| **Hypertension** | 94% | 0.5 | 94% | 95% |
| **PISA (mm^2^)** | 483 (532) | 0.33 | 191 (210) | 518 (547) |
| **CRP (mg/L)** | 7.4 (12.8) | 0.8 | 7.2 (14.0) | 7.5 (12.7) |
| **Total serum FLC concentration** | 108 (187) | 0.8 | 83 (44) | 112 (198) |
| **Isoprostane (pg/ml)** | 26 (20) | 26.8 | 20 (15) | 26 (20) |
| **Protein carbonyls (nmol/mg of protein)** | 1.2 (0.7) | 25.9 | 1.04 (0.7) | 1.2 (0.7) |
| **Currently employed** | 33% | 0.5 | 54% | 31% |
| **Highest Educational Qualification**  None  GCSE  NVQ  GCE A-Level  UG  PG | 40%  24%  8%  9%  13%  7% | 1.6 | 19%  27%  5%  17%  19%  13% | 42%  24%  9%  7%  12%  6% |

GCSE- General Certificate of Secondary Education (aged approximately 16); NVQ- National Vocal Qualification (aged approximately 16–18); GCE A-Level- General Certificate of Education Advanced Level (aged approximately 18); UG- Undergraduate; PG- Postgraduate

Tonetti, M. S., H. Greenwell and K. S. Kornman (2018). "Staging and grading of periodontitis: Framework and proposal of a new classification and case definition." Journal of Clinical Periodontology 45(S20): S149-S161.
